# The Spider Effect: Morphological and Orienting Classification of Microglia in Response to Stimuli in Vivo

**DOI:** 10.1371/journal.pone.0030763

**Published:** 2012-02-21

**Authors:** Rahul A. Jonas, Ti-Fei Yuan, Yu-Xiang Liang, Jost B. Jonas, David K. C. Tay, Rutledge G. Ellis-Behnke

**Affiliations:** 1 Department of Ophthalmology, Medical Faculty Mannheim of the Ruprecht-Karls-University Heidelberg, Mannheim, Germany; 2 Nanomedicine Translational Think Tank, Medical Faculty Mannheim of the Ruprecht-Karls-University Heidelberg, Mannheim, Germany; 3 Department of Anatomy, University of Hong Kong Li Ka Shing Faculty of Medicine, Hong Kong SAR, China; 4 State Key Lab of Brain and Cognitive Sciences, University of Hong Kong Li Ka Shing Faculty of Medicine, Hong Kong SAR, China; 5 Department of Brain and Cognitive Sciences, Massachusetts Institute of Technology, Cambridge, Massachusetts, United States of America; National Institute of Health, United States of America

## Abstract

The different morphological stages of microglial activation have not yet been described in detail. We transected the olfactory bulb of rats and examined the activation of the microglial system histologically. Six stages of bidirectional microglial activation (A) and deactivation (R) were observed: from stage 1A to 6A, the cell body size increased, the cell process number decreased, and the cell processes retracted and thickened, orienting toward the direction of the injury site; until stage 6A, when all processes disappeared. In contrast, in deactivation stages 6R to 1R, the microglia returned to the original site exhibiting a stepwise retransformation to the original morphology. Thin highly branched processes re-formed in stage 1R, similar to those in stage 1A. This reverse transformation mirrored the forward transformation except in stages 6R to 1R: cells showed multiple nuclei which were slowly absorbed. Our findings support a morphologically defined stepwise activation and deactivation of microglia cells.

## Introduction

The blood brain barrier creates an immunologically privileged environment in the brain by limiting the ability of the systemic immune system to remove infections and debris from inside of the brain. Within the central nervous system (CNS), the function of the extracerebral or systemic immune system is taken over by a group of cells called microglia [1], [2], [3], [4], [5]. These cells function in a similar way to how the immune system functions outside of the central nervous system. Despite numerous studies on the action of activated microglia cells [1], [2], [3], [4], [5], , the developmental stages from a resting inactive microglia cell to a fully activated microglia cell have not yet been fully described histologically. Present throughout the CNS and the spinal cord, white matter has fewer microglia cells than grey matter. Microglia cells that are found near blood vessels seem to lose their ramification and become more amoeboid. The amount of microglia is not yet clear. It has been suggested that the population of microglia cells constitutes about 10% [11] to 20% [12], [13] of all cells in the CNS, or about 100 to 200 billion cells depending on the condition of the system [14]. Microglia are activated by pathogens and injured neurons, along with a host of other factors/signals that pose a potential threat to the CNS [13]. Viral, fungal and bacterial structures, complement factors, antibodies, chemokines, cytokines and abnormal endogenous proteins are sensed by the microglial receptors and are responsible for the microglial activation [5], [6], [7], [8]. Since microglia cells are able to sense inflammation, and are the chemical modulators of the local environment [5], [6], [7], [8], [9], it was thought that as soon as inflammation was sensed the microglia became activated and transformed into macrophages. Microglia have also been believed to be neuroprotective [15]. This was first thought to be true only during times of stress and injury; however, at rest the microglia appear to spread out in a grid that allows for sensing the environment without direct cell-cell contact. Any chemotactic change in the environment signals the migration of microglia to sites of injury [16], [17].

**Figure 1:**
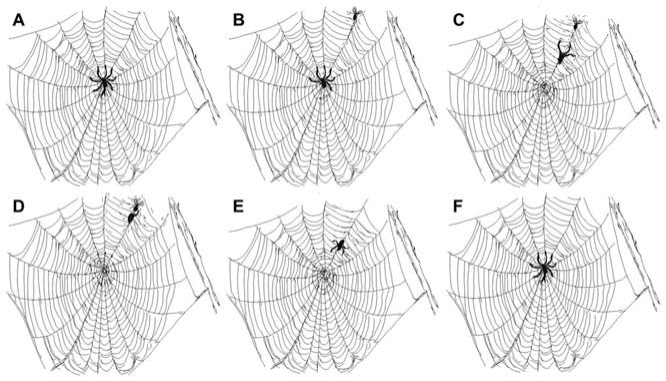
Drawing of a spider web at each stage of the spider behavior. (A): The spider is in the middle of the web waiting for prey to hit the web; it will sense the vibration. (B): The spider senses the fly hitting the web and begins to withdraw its legs and move in the direction of the vibration. The spider is showing directional orientation toward the fly. (C): The spider has left the center of the web and is moving along the primary tension member of the web in the direction of the fly. (D): The spider comes in contact with the fly, seizes the fly, wraps it or eats it. (E): When the fly is consumed the spider starts the return to the center of the web. Again, the orientation of the spider is away from the original disturbance and toward the center of the web. (F): The spider, back at the center of the web, has re-extended its legs to the primary tension members of the web waiting until for another disturbance, or a new meal. The direction of the action is driven by a vibration disturbance that is sensed in the legs of the spider. Microglia appear to use their processes as spider legs and move to the injured site.

Microglia cells resemble spiders: at rest, sitting on their webs, waiting for prey; when alerted (activated), moving toward, capturing and eating prey; afterward, returning to their resting place (Fig. 1). The spider *Xygiella x-notata* lives in huge colonies where the slightest difference in weight on the surface of their net can be detected: ranging from 0.4 mg to 0.05 mg, in extreme cases [8], [9], [10]. The spiders then migrate to the site of potential food. Their wheel-shaped webs allow for this kind of food detection. The spiders sit on the main strings and as they feel their prey become entangled in the web they move toward it and devour it. Similar behavior is seen with microglia. There is evidence that microglia can sense and react to the stimuli [17]. It has been shown that purines can induce chemotactic migration of cultured microglia [17]. Microglia cells sit at the center of their ‘web’ with a foot on each of the tension strings- in this case it can be a chemical signal, a physical deformation, or a combination of both - in order to sense the vibration of disturbances caused at a distance. When a change is sensed, microglia cells retract their processes and move in the direction of that disturbance.

The purpose of our study was to explore the modulation and transformation of microglia cells: from a resting stage to a fully activated stage and returning to rest. We addressed the following questions: how do the cells change morphologically during activation? Is this a one-way route to macrophage? Will the cells return to the site and re-establish the network? After injury, do the cells orient in a direction? If so, can they be used to assess the stage of inflammation? Are the microglia cells replaced through cell division or do they return to the adjacent tissue after they have phagocytosed debris? Here we show a new morphological and orienting classification system for microglia in their activation stages after focal injury.

## Materials and Methods

### Ethics statement

All experiments were carried out in accordance with the ethical standards as described in the Society for Neuroscience guidelines and the National Institutes of Health Guide for the Care and Use of Laboratory Animals (NIH publications No 80-23; revised 1998). Formal approval to conduct the experiments for this study was obtained from the Committee on the Use of Live Animals in Teaching and Research (CULATR) at the University of Hong Kong Faculty of Medicine (CULATR approval number 1581-07). The minimal number of rats necessary to produce reliable scientific data was used and all efforts were made to minimize animal suffering.

The experimental study included adult Sprague-Dawley rats in which the olfactory bulb was transected as standardized model for a stimulus to activate cerebral microglia cells. For the surgery, rats were anesthetized with a mixture of ketamine (80 mg/kg, i.p.) and xylazine (8 mg/kg, i.p.). The olfactory bulb was exposed bilateral by removing the overlying skull. Care was taken to cut the olfactory bulb caudal to the bulb's enlargement. To ensure a complete transection, the knife was inserted through the midline of the skull and it was moved from the midline to the lateral edge of the olfactory tract in one continuous movement. During that movement, contact with the underling skull was maintained. To prevent damage to the venous sinus, the tip of the knife slightly touched the bottom of the brain without penetration of the dura. This procedure was modified from several previous studies in an attempt to isolate the olfactory bulb from the other parts of the brain [8], [9], [10], [18], [19]. The wound cleft was filled with saline using a self-constructed hydraulic pressure injection system with a syringe [8], [9], [10],[18]. Care was taken to slowly inject the saline. After the injection, the overlying scalp was re-sutured and the animals were placed in a warm place for recovery before returning them to their home cages. The animals were sacrificed at 2 days (n = 6 animals), at one week (n = 6 animals) or at two weeks (n = 6 animals) after the intervention. Four normal controls were used for baseline cell counts. All animal procedures were carried out in accordance with The University of Hong Kong guidelines and rules of animal care committee in Hong Kong.

At 2, 7 and 14 days after injury, the animals were sacrificed with sodium pentobarbital (160 mg/kg, i.p.) and perfused transcardially with 0.9% saline, followed by 4% paraformaldehyde in 0.1 M phosphate buffer. Brains were dissected and postfixed in 4% paraformaldehyde overnight at 4°C. Brains were placed in 30% sucrose until they sank at 4°C for cryoprotection. After embedding the blocked brains in OCT medium a freezing cryostat was used for sectioning at 15 microns (Leica CM1900, Leica Microsystems GmbH, Wetzlar, Germany). Sagittal sections of the brains were placed on pre-coated slides and processed for immunohistochemistry. Briefly, the sections were first pre-blocked in a solution of 0.3% triton and 10% goat serum in 0.01 M phosphate buffer solution at room temperature for one hour, and then the primary antibody solution overnight at 4°C before secondary labeling with the corresponding fluorescent-labeled antibody (Invitrogen, Life Technologies, Carlsbad, CA, USA) was carried out at room temperature for two hours. Each step was preceded by three washes in phosphate buffer solution for 5 minutes each. Antibodies and dilutions used include ionized calcium binding adaptor molecule 1 (Iba-1) rabbit anti-Iba-1 polyclonal antibody (1∶500) (Wako Pure Chemical Industries, Osaka, Japan) for microglia, mouse anti-ED1 (Serotec, 1∶500) for macrophages, rat-anti-BrdU (Abcam, 1∶1000) for cell proliferationand Alexa-conjugated, goat anti-rabbit 568 IgG antibody and goat anti-mouse 488 (Invitrogen, Life Technologies, Carlsbad, CA, USA). The slides were then cover slipped with Dako fluorescent mounting medium with DAPI. All images were taken under a 20× or 40× objective with a Carl Zeiss Fluor microscope (Carl Zeiss Inc. Oberkochen, Germany) fitted with a spot camera. All illumination levels were fixed during image acquisition. The BrdU immunohistochemistry required denaturation of the DNA and sometimes tissue penetration enhancement, with HCl (2 N HCl 37°C 30 minutes or at room temperature 1 hour; 1 N HCl) or DNAase treatment and citrate buffer boiling (0.01 M pH6.0 citrate buffer, 95°C 30 min)/enzyme digestion (0.05% trypsin 37°C 10 min, then room temperature 10 min), respectively. We have found that a higher concentrated HCl treatment (4 N HCl room temperature 20 min) was sufficient to stain all cells with BrdU without the need of citrate buffer boiling or enzyme digestion, which may take some sections off the slides. The Iba-1 protein was resistant to the HCL treatment and enabled co labeling without the need to removal of cover slips and a second immunohistochemistry step for Iba-1. Imaging of the z-axis was taken under a 63× objective with a Zeiss LSM 510 META multiphoton microscope to enable localization of labels and cell walls.

**Figure 2:**
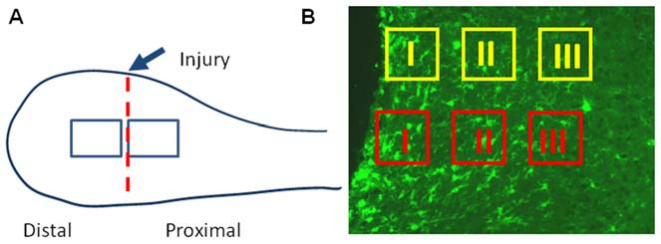
Drawing of the olfactory bulb (A) and the location of the injury and the sampling area. (B): Photomicrograph of the sagittal section of olfactory bulb. The sample grid used for every animal includes both the yellow and red areas and were further broken down into Locations I, II, and III. This was to show the movement and depletion of the microglia from these areas and their subsequent return. Sample grid: 150 µm.

The acquired images were used for categorization and quantification. All cell counting and assessment was performed in a double masked manner. First, each of the acquired images was relabeled with a random code and then the images were passed to a naive member of the research team to fix the 6 counting areas on the images. Then the images were passed to another individual to categorize and count the quantity of each category. Microglia cells were counted in six square areas of 150×150 µm^2^ per slide. The first square was placed at the edge of the injury site and the two subsequent squares had an inter-square distance of 50 µm. Two series of three sampling areas were chosen per photo (Fig. 2 A). Cells were to counted if they fell inside the sampling squares; cells that fell on the top or left sides of the sampling squares were not counted, while cells falling on the bottom and right sides of the sampling square were counted. Since the surgery transected the entire olfactory bulb, it created a distal portion and a proximal portion of the bulb. Distal refers to the section furthest from the brain in the rostral direction while proximal refers to the area closest to the brain. For this study we use the proximal portion of the transected olfactory bulb. This was to avoid the variation of reduced blood flow to the distal portion of the bulb.

At each time point of follow-up (2, 7 and 14 days after injury), we assessed the morphology and orientation of the activated microglial cells at each sampling area in a distance of zero µm (area I), 200 µm (area II) and 400 µm (area III) from the injury site (Fig. 2). In the morphological assessment, we first examined the stage of microglia activation according to the cell morphology. It was based on the criteria of cell body size and shape, number of cell processes, length of each process, orientation of the cell processes, number of branches and length of the branches. We then assigned a type number of “1” to “6” where “6” was a fully activated macrophage. The cell bodies were measured to determine the variation between the cell types. The number of microglia cells for each category was converted to density (cells per mm^2^) to normalize the counts. This was then converted to percent of the total for each sampling area and time point. A subset of images was taken for analysis to localize the nuclei within the cell walls for DAPI, iba-1 and ED-1 staining. A second set of images was taken with BrdU labeled sections to assess cell proliferation. For the assessment of the orientation of the microglial cells, we made a determination based on the orientation (direction) of the majority of the cell processes by assigning the direction based on the orientation of the side processes in relation to the injury. There was prior evidence that the microglial processes direct towards the injury site [8], [9], [10], [18]. If the majority of the cell processes extended in the direction of the injury then the cells were labeled advancing (A) microglia while the cells with the majority of their processes oriented in the opposite direction to the injury site were termed returning (R). Cells with undetermined orientation were labeled “undetermined” or “U”.

## Results

### Characteristics of “advancing” activated microglia

**Figure 3:**
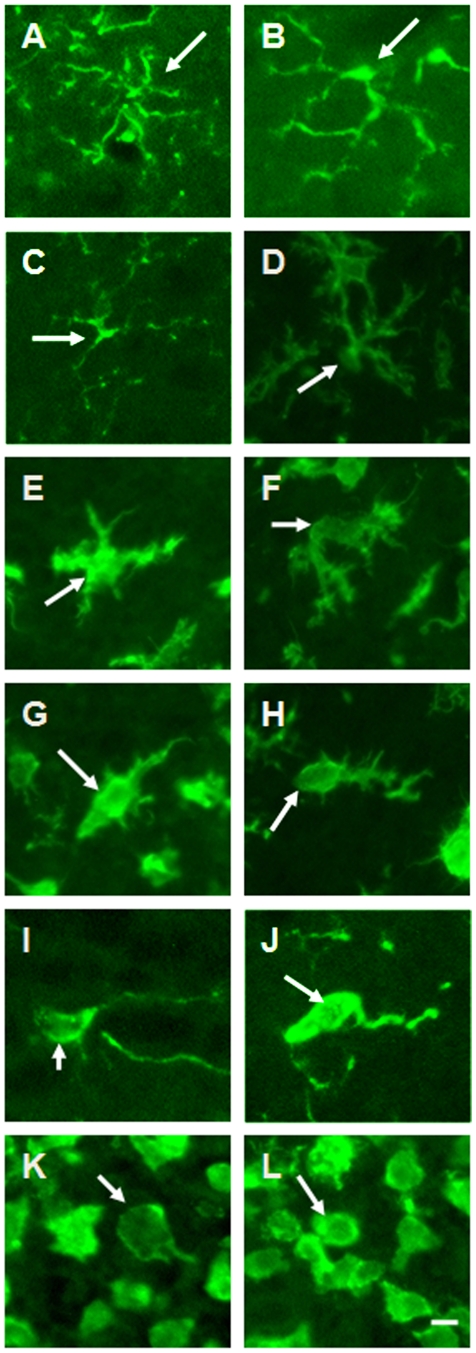
Photographs of microglia cells in the olfactory bulb 2 days after a standardized transection of the olfactory bulb sectioned and stained with Iba-1. These photomicrographs are showing the cells advancing. (A and B): Stage 1A, a microglia cell has processes that are ramified and spread out, with a small soma. (C–D): Stage 2A, the soma has increased in size to approximately 1.5–2 times the soma diameter of a stage 1A cell. The cell processes have started to retreat and the branches next to the cell soma are thickened. (E–F): Stage 3A, the cell soma diameter is enlarged to 2–3 times the soma diameter of stage 1A. All cell processes have retracted and thickened. (G–H): Stage 4A, the soma diameter is 3–4 times larger than the stage 1 soma; all thin cell processes have completely retracted and only the thick cell branches remain. (I–J): Stage 5A, soma diameter is 5 times larger than the soma diameter in stage 1; the thick process is replaced by a thin process oriented in the direction of the cell movement. All branches are gone. (K–L): Stage 6A shows the transformation from microglia to macrophage. The microglia cell has a large round morphology with a large soma, with one short or no processes. (Scale bar: 10 µm).

**Figure 4:**
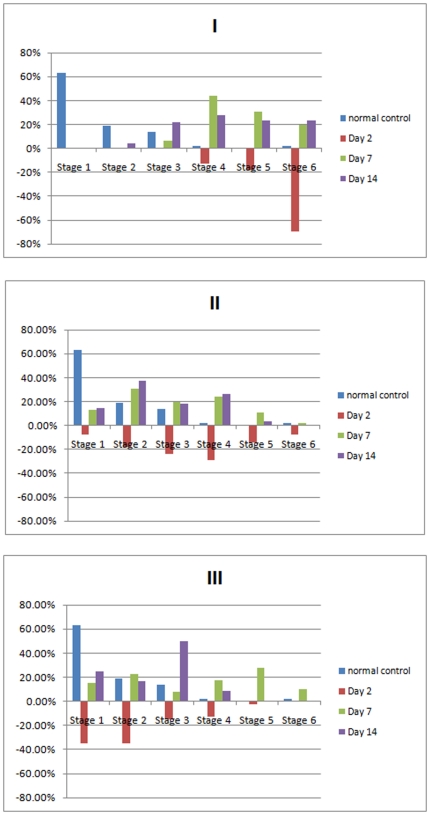
Relative microglial cell density in regions at zero µm (I), 200 µm (II) and 400 µm (III) from the injury site at 2, 7 and 14 days after experimental transection of the olfactory bulb, stratified into 6 stages according to the morphology of resting versus activated cells.

**Figure 5:**
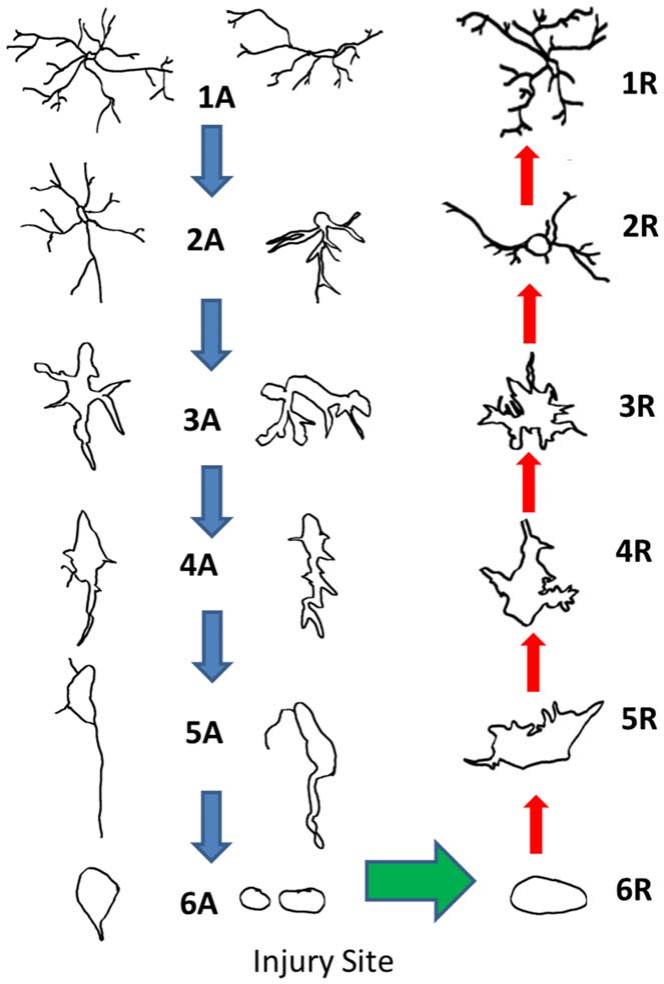
The drawing are tracings of the resting activated microglia starting in resting stage 1A, continue to an activated stage 6A (macrophage stage), transform to stage 6R (multinuclear cells), and then return to a resting stage 1R. The blue arrows indicate the increase in activation and the green arrow indicate the transition from activated to returning. This demarcation appears to be the point that the macrophages consume other cells and debris and are digesting or carrying it away from the event horizon. Note that the cells of stage 6R to stage 3R appear larger than the cells of other stages. This is possibly due to the increased number of nuclei that they have consumed. Some of these nuclei and cell debris appeared to have been transferred to other macrophages.

Table 1 shows a summary of all of the morphological features that distinguished all of the stages of activation of microglia. In stage 1A, the first stage of activation, the microglia cells had a small soma, with widely spread ramified processes and which showed no directionality (Fig. 3 A, B). In stage 2A, the soma increased in size to approximately 1.5 to 2 times the diameter of a stage 1A cell. The cell processes, which were still ramified, retreated on the side away from the injury; and the remaining branches, closest to the cell soma, began to thicken (Fig. 3 C, D) giving the appearance that the cell was orienting toward the injury site. In stage 3A, the cell soma diameter had enlarged to 2 to 3 times the soma diameter at stage 1A and most cell processes had retracted and thickened (Fig. 3 E, F). There were usually two or more thick processes and several thin ones oriented in the direction of the injury. The long thin branches were replaced by short thick branches as they retracted. The processes had disappeared on the side of the cell away from the injury. In stage 4A, the soma diameter was 3 to 4 times larger than that in stage 1A; all long thin processes had completely retracted and only the thick branches remained (Fig. 3 G, H). Usually there was one prominent process oriented in the direction of the injury site with several smaller processes at a 90° angle to the injury site. These processes usually were shorter than 25 percent of the soma size. In stage 5A, the soma diameter was enlarged to 5 times the cell size in stage 1A; the prominent thick process had thinned and appeared to be oriented in the direction of the injury site and were as long as 75 µm (Fig. 3 I, J). The emergence of the ED1 signal also appeared and continued through stage 5R. Stage 6A was characterized as a fully activated microglia cell with a macrophage character (Fig. 3 K, L). The long process was very short: 50% of the soma diameter or completely gone. The cell was spheroid or spherical in shape and the soma had grown in size to 14±2 microns. The cell was at the edge of the injury site, or within 50 microns of the edge of the damaged tissue. In Graph I, II and III, the advancing microglia cells are shown in red in the negative region of the graph show that they are migrating toward the injury site (Fig. 4). The regressing microglia cells are shown in green and purple described in detail later. The full range of activation to complete deactivation is shown in tracings of selected prototypic cells from the tissue for each stage (Fig. 5).

**Table 1:** List of characteristics of each stage of microglial activation and de-activation.

Cell Stage	Soma Size (µm)	Process Number	Process Diameter (µm)	Branches Number	Process Length/Soma Diameter
1A	2.5–5.3	4+	<1	Compound Long	>10×
2A	5.0–6.8	3–4	1–2	Compound Short	>4×
3A	6.5–10.0	4+	2.5–3.6	Short	1–3×
4A	8.3–10.7	2–4	2.5–5	Short	1–2×
5A	10.2–15.2	1–2	<1.5	None	>2×
6A	12.1–16.6	1-none	<1.5	None	<1×
6R	12.0–18.4	1-none	<1.5	None	<1×
5R	11.4–15.2	1–2	1–2.5	None	1×
4R	8.3–11.5	2–4	2.5–5	Short	1–2×
3R	6.5–11.3	4+	2.5–3.6	Short	1–3×
2R	5.0–6.8	3–4	1–2	Compound short	>4×
1R	2.5–5.3	4+	<1	Compound Long	>10×

The cell stage was defined by soma size, process number and process diameter, number of branches and process length in relation to the soma diameter. The data for the table was the result of measuring between 150–200 cells per stage.

### Characteristics of “returning” activated microglia

**Figure 6:**
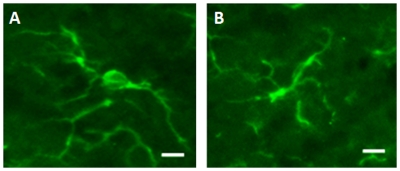
Photographs of microglia cells in the olfactory bulb 7 days after a standardized transection of the olfactory bulb sectioned and stained with IBA-1 (green). (A): Stage 2R, the soma decreased in size to about 1.5–2 times the soma diameter of a stage 1A cell; the cell processes lengthened and the cell lost its directional orientation. Branches were clearly prominent on the processes. (B): Stage 1R, the microglia cell had a small soma with processes that were ramified and spread. The cell was back to its original location and separated from the other neighboring microglia (Scale bars: 10 µm).

Stage 6R was characterized as a multinucleated cell because of cell debris phagocytosis. Most cells had a prominent ED1 signal indicating they had transformed to macrophages but continued to retain the Iba-1 signal. The location of these cells was at the injury interface; however, there were a few exceptions. A small population of the cells with prominent ED1 staining appeared adjacent to dead or dying cells in the olfactory bulb. As the cells acquired more than three nuclei, the macrophages appeared to change direction and started to reorient away from the injury site. Stage 5R cells had more than three nuclei and extended a thin process away from the injury site. Some of these cells continued to retain the ED1 signal; however, it appeared that the signal was quickly lost. In stage 4R, at some distance to the injury site, the cell extended 1 to 3 thick processes oriented away from the injury site. All evidence of ED1 labeling was absent while Iba-1 labeling remained. In stage 3R the microglia extended 4 to 7 large short processes oriented away from the injury site. There were also one to two thin, short processes that were extended in the direction of the injury site. Many cells still contained multiple nuclei. In stage 2R, the processes became thinner and 4 to 5 times longer than the soma. Though most of the processes were oriented away from the injury site there were one or two oriented in the direction of the injury site. Finally, in stage 1R the cell had returned to the inactivated state and extended the processes in all directions (Fig. 6). Stages 1A and 1R morphologically appeared to be the same.

### Changes in density

**Figure 7:**
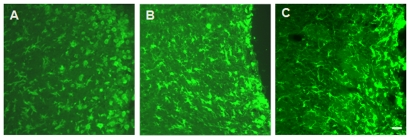
Photomicrographs of sagittal sections of the olfactory bulb with the experimental incision injury site to the right. (A): Day 2 post injury. Note the accumulation of small round cells at the injury site (at the right edge of photo); the orientation of the cell was in direction of the injury site. (B): Day 7 post injury. The small round cell bodies partially disappeared and the cell orientation was away from the injury site. The cells appeared to be returning to the tissue. (C): Day 14 post injury. There were only a few areas that still had high concentrations of round microglia cell bodies/macrophages; the cells were oriented away from the injury site and some highly branched cells were present distant to the injury site. The green was Iba-1 with an Alexa dye.

Overall microglial activation followed the induced injury to the olfactory bulb (Fig. 7). The level of activation was evident at the second day after the injury (Fig. 7A) with spherical cells clustered close to the injury site. The Iba-1 staining showed fully activated macrophages next to the injury site in stages 4A to 6A, while the cells located distant to the injury site were activated and showed an orientation in direction to the injury site. By day 7, the level of activation decreased with the majority of cells in stages 6R to 4R (Fig. 7B). At 14 days after the injury, some cells in stage 6R remained at the edge of the injury site. The orientation of the cells had changed, pointing in the direction away from the injury site for stages 5R to 1R. The cells of stage 1R were located in the region distant to the injury site.

**Figure 8:**
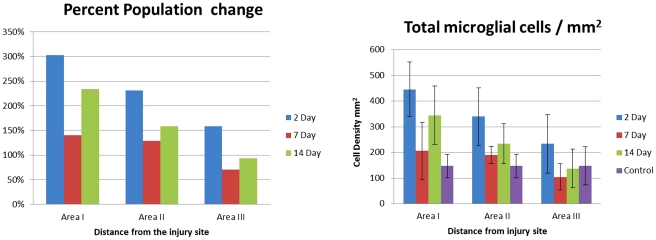
Microglial cell density (cells/mm^2^) at 2, 7 and 14 days after experimental transection of the olfactory bulb in regions at zero µm, 200 µm and 400 µm from the injury site.

The mean number of microglia cell bodies in the olfactory bulb in the untreated controls was 147±10 cells/mm^2^. Stratified into the different stages, the mean density of microglia cells for each stage 1, 2, 3, 4, 5 and 6 was 82±5, 20±2, 18±2, 9±1, 8±1, and 7±1 cells/mm^2^ respectively. In the study group, from the time point at 2 days after injury to the time point of 14 days after injury, we overall detected a progressive depletion of microglia cells in the sampling regions II and III; while, at the same time, sampling region I showed a significant increase in the density of microglia cells. At the second day after injury, at the injury interface (area I), the microglia cell density for the saline injected animal were 0, 0, 18±1, 88±4, 40±3, 303±14, totaling 451±105 microglia cell bodies/mm^2^. This represented a three times increase in the density of microglia/macrophages from normal tissue (Fig. 8). These numbers decreased towards day 7 after injury, and then re-increased at 14 day after injury (Fig. 8).

Stratified by the stages of the cells, the histological slides obtained at 2 days after injury showed closest to the injury site mostly cells in stages 4A to 6A, while cells in stages 1A, 2A and 3A were mostly absent (Fig. 4). By day 7, the slides showed predominantly microglia cells in stages of 6R, 5R, 4R to 3R (Fig. 4). By day 14, cells in stages 6R through stage 2R were present. A multivariate analysis, with the time point as a dependent parameter, and cell density of the 6 stages and location (area I to III) as independent parameters, revealed that the relative cell density of stage 4 (*P* = 0.01) and stage 6 (*P* = 0.002) decreased significantly with increasing time points. After adjustment for location, the relative density of cells in stage 4 and stage 6 decreased significantly with advancing time.

Applying BrdU at the time of olfactory bulb transection revealed that at 2 days after the injury 37% of the microglial cells to be BrdU positive. At 7 days after injury that figure was reduced to 3.5%, when BrdU was added at day 6. There were some BrdU-positive cells that returned to the original stage 1 morphology.

### Distance from injury site

**Figure 9:**
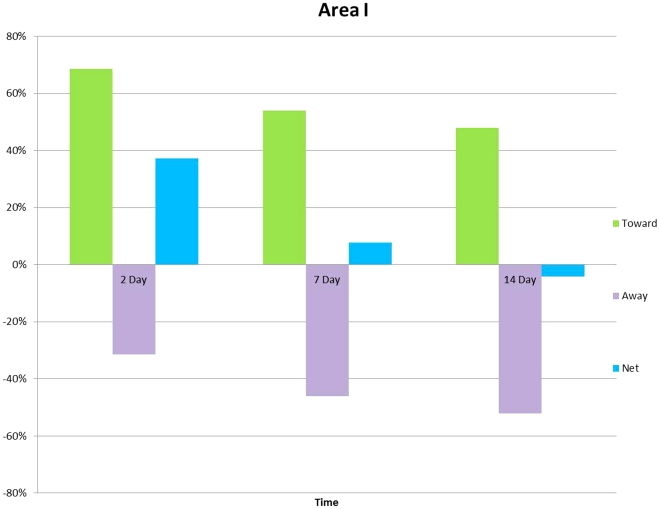
Graph showing the distribution of microglial cell processes oriented toward (green bars, UP) or away (purple bars, Down) from an experimental transection site of the olfactory bulb the cut at 2, 7, and 14 days after the injury. The blue bar is the net direction of the processes.

The distance from the injury site affected the predominant morphology or stage of the microglial cells. There was a U-shaped distribution of the density of the microglia cells stratified by the stages. The highest density was found next to the injury site and at the location of 200 µm distant to the injury (location II). At day 2, at 200 microns (location II) from the injury site, only 45% of the microglia cells were present; and at 400 microns (location III) that number increased to 77% of the injury site. In location II, stages 1A through 6A of microglia were present and were oriented in the direction of the injury site (Fig. 9). A multivariate analysis, with the sample area (area I–III) as a dependent variable and the cell density of stages 1–6 and time as independent variables, showed an association between a higher concentration of cells of stage 1 (*P* = 0.06) with increasing area number (i.e., more cells of stage 1 in area III); and an inverse association between the cell density of stage 3 (*P* = 0.008), stage 4 (*P* = 0.005) and stage *6* (P<0.001) with the area number, i.e. densities of cells in stages 3, 4, and 6 were higher in area I compared with area III.

### Orientation of processes

**Figure 10:**
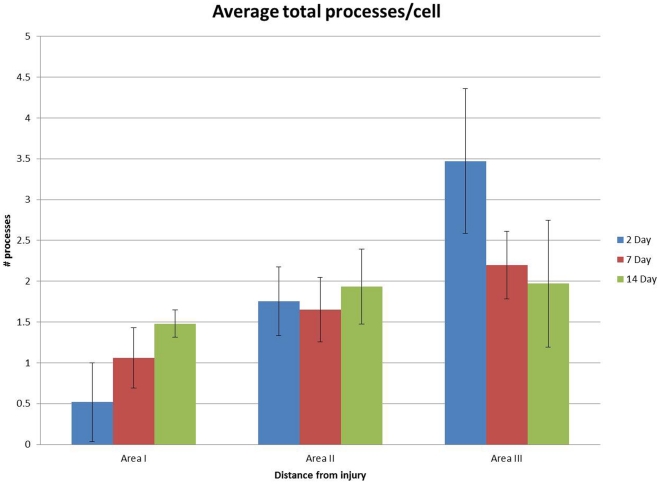
Graph showing the mean number of processes per microglial cell at zero µm (area I), 200 µm (area II) and 400 µm (area III) from the injury site at 2, 7 and 14 days after experimental transection of the olfactory bulb. Control animal had an average of 5.8 processes per cell. The bars show the standard deviation.

The mean number of processes for each of the time points and location were: Area I; Day 2, 7, 14: 0.5, 1.0, 1.4 processes per cell. The orientation of these processes was in 68% of the cells toward the injury site, and in 31% of the cells away from the cut. By day 14, the processes of 47% of the cells were orientated toward, and the processes of 52% of the cells were orientated away from the cut. For Area II, the processes of 68% of the cells were orientated toward the transection site, and the processes of 35% of the cells were orientated away from the injury site. By day 14, the processes of 52% of cells were orientated away from the injury site, and the processes of 47% of the cells were orientated toward the transection site. The mean number of processes per cell in Area II was 1.7, 1.6, and 1.9 for 2, 7 and 14 days after the injury, respectively (Fig. 10). In Area III, the mean number of processes per cell was 3.4, 2.1 and 1.9 at days 2, 7, and 14 respectively (Fig. 10). In area I, the number of processes per cell was relatively low as compared with areas II and III, and it increased significantly (*P* = 0.009) from day 2 to day 14 (Fig. 10)

## Discussion

### Morphological classification of activated microglia cells

Using the model of standard injury to the olfactory bulb, we classified microglia cells into 6 morphological stages and further added the designation of ‘A’ for advancing and ‘R’ for returning. The third designation was an ‘U’ for undetermined when the orientation of the cell could not clearly be detected. Cells designated with “U” were mostly seen in normal controls. The classification began with stage 1A with a small soma and widely spread ramified processes and proceeded to stage 6A of a fully activated microglia cell with a large, round soma and few thin, if any, processes emerging from the soma. With increasing time after injury, the classification reversed from stage 6R to stage 1R where the highly ramified microglia cells were again equidistant from its neighbors. Stages 6A and 6B were differentiated by the presence of phagocytosis and multinuclear cells in stage 6R. An additional possibility to explain the presence of multinucleated cells in stage 6 was that some microglia cells fused with each other. In a study by Petersen and colleagues, phagocytosed nuclei were transferred between microglial cells [20].

### Bidirectional orientation

Using histological criteria, activated microglia were differentiated along with the recognition that the process was bidirectional with 6 progressing stages, and by 7 days post injury, 6 regressing stages. The turning point in the orientation of the microglia cells from progressing to regressing occurred at approximately 3 days after the experimental injury.

In the progressing stages, the microglial cells had a clear directionality; the cells were morphologically oriented in the direction of the injury site. The processes appeared to be retracted on the side opposite the injury site, but remained extended on the side toward the direction of the injury site. Immunohistochemistry showed that at stage 5A the cells were ED1 positive along with Iba-1. As the cells became more densely concentrated close to the site of injury, they became less ramified and usually had only one process just prior to entering the area of damage. The stages 6A or 6R microglia/macrophage also had an ED1 signal along with Iba-1. It appeared as if these were the cells of stage 6R getting ready to phagocytose dead or dying cells along with debris. After phagocytosis of a sufficient amount of debris, the activated microglial cells appeared to change orientation away from the injury site. It has remained unknown, how much debris a transformed macrophage or microglia of stage 6R was able to phagocytose.

**Figure 11:**
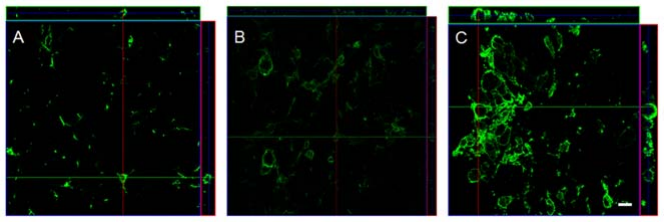
Multiphoton Z-Stack images of microglia cells immunohistochemically stained by Iba-1 to show the cell bodies and cell processes. The panels show a Z-Stack of images while the panels to the right and top demonstrate the level of the image in the stack. (A): partially activated microglia with a few activated cells. The cross hairs show the location of the cell and the upper and right panels show the depth of the cell. (B): activated microglial cells as by increased size and more circular shape size. (C): fully activated microglia with several microglia cells in stage 5R. Note: the cells cover the full thickness of the histological slide and although some cells are located close to each other, they can clearly delineated from each other by the Iba-1 label in the cell walls. Scale bar: 10 µm.

In the regressing stages, microglial cells of stage 5R had processes oriented away from the injury site, deemed as retreating, and showed clearly visible multiple nuclei. To prevent an artifact, we confirmed the multinuclear status of these cells by using thin histological sections to avoid an overlapping of cells. To ensure that the nuclei were located only in one cell, we used confocal imaging in a second step and examined a Z-Stack of images using the Iba-1 signal to delineate the cell walls [21]. As shown in Figure 11, the confocal images revealed a clear demarcation of the cellular boundaries, but could not rule out the possibility that the nuclei were being passed from one cell to another. Between the microglial cell stages 3R and 2R, the microglia cells lost the additional nuclei so that from stage onwards, all the cells were mononuclear. The loss of the multinuclear status was possibly due to the breakdown of the debris, or resulted from a cell apoptosis. Since we did not detect any tunnel positive and Iba-1 positive staining at later time points, one may infer that the microglia may not have died but may have digested the phagocytosed additional nuclei. We detected some BrdU positive cells that appeared to have taken their original morphology of stage 1. As the microglia regressed to stages 2R and 1R, they lost the orientation bias and re-extended their processes, becoming indistinguishable from the stage 1A microglia. The ability of the previously activated microglia cells to regress to their original shape and location suggest that they might have memory, which has been discussed previously [22].

### Distance from injury site

Through the sample grid which extended to 550 µm from the injury site we detected a distance-dependent transformation of the microglia. In the furthest sampling area (area III) at the second day after injury, the predominant cell stage was 1A to 3A (Fig. 4), while there was a paucity of cells in stage 5A and 6A. In contrast, in the area closest to the injury, the majority of the microglial cells were in stage 5 and 6 (Fig. 4). Later at seven days after the injury in area III distant to the site of injury, the density of cells of stage 6R had markedly increased. It indicated that the transformation of cells from stage 1 to stage 5 and 6 had taken place in a distance dependent, and therefore presumably concentration dependent, manner. It would agree with the studies on ATP/ADP dependent signals for homing of microglial cells to the site of injury [21].

### Concentration of activated microglial cells of stage 6

Comparing the three study groups with each other revealed that at the second day after the intervention, the concentration of stage 6 cells increased significantly to 69% of the total microglia/macrophage population at 2 days after the injury, and decreased at 1 and 2 weeks after baseline to 20% and 24% of the total microglia/macrophage population. By the second week post injury, 30% of the microglia cells were at stage 3R. This appeared to be a wave of regressing and potentially returning cells to stage 1R. Further time points would have been necessary to follow the cells back to a completely normal control level. The response was proportional to the distance from the injury site with more and higher activated cells in the areas closer to the injury. It may have been due to the inflammatory signals concentration decreasing with increasing distance to the site of injury.

### Multinuclear cells

The primary distinguishing feature between microglia cells of stage 6A and stage 6R was the presence of multiple nuclei. We considered the presence of more than 2 nuclei as a sign to distinguish them from dividing microglia. The second distinguishing feature was the time frame. At 2 days after injury, we detected few cells with 2 nuclei which were BrdU positive. The addition of BrdU was made at the day of the cut in one group, and in a second group at day 6. The two day group showed a marked increase of BrdU signaling early, however at a later time point (day 14) this signal disappeared completely. Another possibility that may be considered is that the multinucleated cells could have been a result of microglia cells fusing with each other. This would create a multinucleated cell; and the clustering seen by others [21] could be a precursor stage for the eventual fusing of the cells. This also may be a way to clean up the area without the debris and without inflammation usually seen outside of the CNS.

### Spider effect

Based on the findings described above, one may consider microglial cells to behave like spiders sitting in the middle of their web. When a spider detects vibration on one of the primary tension components of the web, it moves its legs to the two adjacent strings to localize the vibration. Then the spider retracts its legs from the other members and moves in the direction of the vibration. However to suggest that the legs and the feet of the spider are stationary the entire time would be an oversimplification. They are palpating the web and constantly readjusting. In addition, the farther away the spider is from a source of movement in the web, the weaker the signal. The microglial cells examined in our study may have behaved in a similar manner as spiders do. Based on the predominant morphology of the microglial cells stratified into the 6 stages of activation, the time of follow-up, and the distance to the site of experimental injury performed in our study, it appeared that the microglia retracted their processes after sensing and localizing the damage signal and then moved in the direction of the strongest signal, thus depleting the local area of microglia cells. As the microglia moved to the injury site, they transformed morphologically from stage 1A to stage 6A. In addition, it appeared there that also other cells proliferated and infiltrated the region. The percentage of cells that were positive for both Iba-1and BrdU was 37% of the total microglial population when the BrdU was applied at the time of the transection. This figure was higher than the figure of 25% as found by Wohl and colleagues [23]. When the crisis was over and the damage was repaired, the microglia again transformed morphologically, reversing direction, from stage 6R to 1R, toward their original location, extending their processes and moving to adjust themselves to be located equidistantly to each other. In a similar manner, spiders return to the middle of their web extending their legs back to several of the tension components of the web in an equidistant way and waiting for the next disturbance.

Potential limitations of our study should be mentioned. First, as any experimental study our investigation cannot be taken as prove for a real clinical situation. Second, our study used fixed tissue, which was examined immunohistochemically. Therefore, in contrast to an *in vivo* examination, it was not possible to follow individual microglial cells in their development from a resting “observing” stage (stage “1A”) with an equidistant distribution of cells in the tissue before the experimental injury was set to an activated stage (stage “6A”) of microglial cells moving to the site of injury and phagocytizing tissue debris, before returning to their original location and function (stage “1R”). By comparing, however, the snapshots of the histologic images taken at 2 days, 7 days and 14 days after the experimental injury, the assumption of the development of activation and de-activation through stages “1A” to “6A” and from “6R” back to “1R” appeared deducible. Third, the measurements in our study were based on the staining of the microglial cells. Although the immunohistochemical staining of microglial cells by Iba-1 has been described to be relatively specific and sensitive for microglial cells, there is the possibility that some microglial cells were not stained and that other cells were stained and falsely counted as microglial cells. Fourth, we used the location and direction of the cell processes to assume the direction of the movement of the microglial cells. In vivo, however, the cell body including the processes are three-dimensionally arranged so that the appearance of the microglial cells in the mostly two-dimensional histological slides can only be taken as a surrogate for the three-dimensional structure of the cells and the direction into which their processes point to. In addition, the telltale signs of direction sensitivity and the orientation of the microglial cells resemble that of migrating neurons seen in early development; also in the rostral migratory stream of the olfactory bulb. The data of our study, therefore, suggest, however, cannot definitely prove, that the microglial cells move from their resting location in the vicinity of the site of injury to the cutting edge of the injury in their activated stage and then back to the original location in their then again resting stage. Interestingly, however, our findings support a recent investigation by Ohsawa and Kohsaka who showed that microglia processes orientate in the direction of an acute injury due to a release of ATP (adenosine triphosphate) [24]. Fifth, there were several different labels available for use to differentiate microglia at the various different stages of activation. There were reports in the literature that different stages of activated microglial cells can be differentiated by specific cell surface markers. For example, lectin staining has been used to detect microglia of multiple species [25]. The fluroscence-lectin provided staining of the microglia without the need for a second antibody. The successful application of lectin (especially lectin from *Griffonia simplicifolia* for rat microglia) staining on microglia can be used for staging of the microglial activation *in vivo* as well. However, we found that Iba-1 was similar to the lectin staining but did not stain the vasculature. We also used ED1 because this allowed for the staging of activated macrophages. When the two were co-localized we found that the combination of Iba-1 and ED1 was a better choice to stage the microglia. When both ED1 and Iba-1 were seen, the microglia cells were at stage 5R and the ED1 signal remained with the microglia until stage 5R. When stage 4R was reached, the ED1 signal was lost.

In an experimental study including the standardized transection of the olfactory bulb, we developed a classification system for activated microglial cells. By assessing the shape of the microglia cells and reading the direction of the microglia migration, that classification system can be used to assess the effectiveness, duration, and distribution of inflammation as well as the direction, extent of the injury site and the time lapse after injury has occurred. The staging of the microglial activation and eventual deactivation may lead to a better understanding of some types of inflammatory of the CNS, including the question of why the microglia will take on a certain type of morphology in one disease and a different one in another disease. The classification system may serve as a physical biomarker for future therapies and staging of diseases noninvasively.

Since the microglial morphology changes in response to the environment we may be able to use microglia as a way to measure the stage of a disease. This system also raises additional questions as to whether the multinucleated transition of the microglia cells is due to cell fusion of microglia that starts with clustering. This may send a signal that the area is clear, to initiate the return to their original position. There is one additional possibility: that as the cells move to the area of injury creating an area that is depleted of microglial coverage, this could send the neuronal tissue into a mode of decreased activity, or even shock. When the microglia return and are able to re-establish the normal grid it would allow the neurons to return to a normal level of activity. The population change at each area has a u shaped population density. Though this is does not appear significant it may represent a need to extend anti-inflammatories longer than the typical 7 days in a clinical setting. This second wave of cells are not stage 5A/R or 6A/R cells however they represent a possible longer term autocrine signaling that may extend the activation period beyond what was previously thought.

The results of our study agree with previous investigations in that we found signs that microglia cells were bidirectional; they did not only cluster and migrate toward an injury site, which resulted in a higher density, but they also appeared to migrate back to their origin, although this was the first it had been seen *in vivo*. Our study also agrees with the study by Carbonell and colleagues on migration of microglia in slice culture [22]. This, along with our data, suggests that the migrating microglia will migrate toward the site of injury much the same way a spider will migrate toward a disturbance in the web.

Our study extends the findings of previous investigations by defining the stages of microglial transformation morphologically from stages 1A to 6A and back from 6R to 1R, with clear definitions based on process numbers, size of processes, orientation and soma size ranges. We have laid out a classification system to standardize the microglia activation levels. This will also allow for staging of inflammation based on cell morphology and activation level. It could give us clues as to how to test for previous exposure to pathogens. Depending on the reaction from a biopsy we may be able to use this for a new morphological biomarker for early detection or even disease progression. Due to the change in morphology it shows that the cell is reacting differentially to different types of attack. This could be due to bacterial invasion, simple inflammation or even the start of cancer. By assessing the activation stage of the microglia, it might be possible to determine the extent of the infection or another underlying disease process noninvasively, looking through the lens of the eye.

In order to determine if this was a robust classification scheme it was given to two colleagues experienced in cytology and histology. We asked them to evaluate images from random sections, without previous training. The two naïve observers came up with a result that varied from each other less than 2% for each stage, except for the transition from 6A to 6R, where the variance was 8% between the subjects. Since there are specific measurements for Stage 1A, there should be no problem in reproducing the results. All of the other stages were based on the numbers of stage 1A. For example, in stage 5A the diameter of the cell was enlarged to 5 times the cell size of stage 1A. The processes were also evaluated and measured. We have continued to use this classification system examining different tissues and it appears to be robust for the examination of ocular tissue and other parts of the brain.
